# Absolute Quantification of Individual Biomass Concentrations in a Methanogenic Coculture

**DOI:** 10.1186/s13568-014-0035-x

**Published:** 2014-04-12

**Authors:** Helena Junicke, Ben Abbas, Joanna Oentoro, Mark van Loosdrecht, Robbert Kleerebezem

**Affiliations:** 1 Department of Biotechnology, Delft University of Technology, Julianalaan 67, Delft, 2628 BC, The Netherlands

**Keywords:** Anaerobic digestion, qPCR, Individual biomass concentration, Biomass specific conversion rates

## Abstract

Identification of individual biomass concentrations is a crucial step towards an improved understanding of anaerobic digestion processes and mixed microbial conversions in general. The knowledge of individual biomass concentrations allows for the calculation of biomass specific conversion rates which form the basis of anaerobic digestion models. Only few attempts addressed the absolute quantification of individual biomass concentrations in methanogenic microbial ecosystems which has so far impaired the calculation of biomass specific conversion rates and thus model validation. This study proposes a quantitative PCR (qPCR) approach for the direct determination of individual biomass concentrations in methanogenic microbial associations by correlating the native qPCR signal (cycle threshold, Ct) to individual biomass concentrations (mg dry matter/L). Unlike existing methods, the proposed approach circumvents error-prone conversion factors that are typically used to convert gene copy numbers or cell concentrations into actual biomass concentrations. The newly developed method was assessed and deemed suitable for the determination of individual biomass concentrations in a defined coculture of *Desulfovibrio sp.* G11 and *Methanospirillum hungatei* JF1. The obtained calibration curves showed high accuracy, indicating that the new approach is well suited for any engineering applications where the knowledge of individual biomass concentrations is required.

## Introduction

Many biotechnological processes rely on the combined action of complex microbial consortia (Kleerebezem and van Loosdrecht [2007]). An important example is the anaerobic digestion process which converts organic residues into biogas, a renewable form of energy containing methane as the primary energy carrier (Chen et al. [2008]; Gujer and Zehnder [1983]).

Anaerobic digestion comprises a series of reaction steps each performed by a specific microbial group of the anaerobic ecosystem (Gavala et al. [2003]; Stams and Plugge [2009]). Due to the interdependence of involved reactions, the overall mechanism is kinetically controlled by the rate limiting reaction step (Griffin et al. [1998]; Lyberatos and Skiadas [1999]; Yu et al. [2005]). Therefore, to improve conversion performance and process control major importance lies in the identification of the factors that govern a well-balanced reaction mechanism (Chen et al. [2008]; Griffin et al. [1998]; Rittmann and McCarty [2001]). To investigate these factors mathematical models such as the Anaerobic Digestion Model No.1 (ADM1) have been developed (Batstone et al. [2002]; Gavala et al. [2003]). Unfortunately, validation of ADM1 and similar models is yet hampered by the lacking information on *individual biomass concentrations* i.e., the biomass concentrations of individual species or different functional groups contained in the microbial community. Only by knowing individual biomass concentrations it is possible to calculate biomass specific rates which form the basis of these models and whose determination is hence required for their evaluation.

In mixed microbial conversions any rate should be normalized to the individual biomass that is associated with it, giving rise to a *biomass specific rate,* q, defined as

(1) $$q=\frac{R}{N_{x}}=\frac{R}{c_{x}*V_{R}},$$

where R denotes the net reaction rate in question, N_x_ the specific biomass amount, c_x_ the specific biomass concentration and V_R_ the reactor volume (Heijnen [2010]). In the general case, dividing by the lumped instead of the individual biomass provides an incorrect description of experimental conditions as certain reactions are performed by specific organisms only and not by the total biomass. A failure to implement this logic into mixed microbial conversion models may result in inaccurate and even false predictions. The measurement of individual biomass is therefore an important step towards an improved theoretical understanding of the anaerobic digestion process and mixed microbial conversions in general.

Only limited research has focused on the determination of individual biomass concentrations in mixed microbial communities. Seitz et al. ([1990]) determined individual biomass concentrations of an anaerobic coculture by phase-contrast microscopy assisted manual cell counting. Nevertheless, this approach is time-intensive and suffers from low accuracy since morphologically similar microorganisms and aggregated cells can hardly be distinguished (Manz et al. [1994]; Wagner et al. [2003]). Emerging molecular techniques such as qPCR, quantitative fluorescence in situ hybridization (qFISH) or pyrosequencing promise to be much faster and more accurate (Coskuner et al. [2005]; Ronaghi and Elahi [2002]; Wagner et al. [2003]; Zhang and Fang [2006]).

Previous studies have used qPCR to analyze microbial community structures and population dynamics in a range of samples from wastewater treatment plants (Hall et al. [2002]; Harms et al. [2003]; Winkler et al. [2012]), anaerobic bioreactors (Shin et al. [2011]; Yu et al. [2006]), activated sludge processes (Hall et al. [2002]; Kim et al. [2011]) and natural habitats (Schippers and Neretin [2006]). Similar applications were covered by qFISH (Albertsen et al. [2012]; Egli et al. [2003]; Juretschko et al. [2002]; Kragelund et al. [2011]) and pyrosequencing (Jaenicke et al. [2011]; Kröber et al. [2009]; Kwon et al. [2010]; Schlüter et al. [2008]; Zhang et al. [2012]). However, only a few attempts addressed the absolute quantification of individual biomass concentrations or the calculation of biomass specific conversion rates.

Harms et al. ([2003]) quantified nitrite-oxidizing bacteria (NOB) and ammonia-oxidizing bacteria (AOB) by means of qPCR and derived cell-specific conversion rates in activated sludge. The native qPCR results (DNA copies/L) were converted to cells/L, cells/g, and percent of biomass using several assumptions. Ahn et al. ([2008]), Cho et al. ([2013]) and Kindaichi et al. ([2006]) used qPCR to determine maximum biomass specific growth rates in nitrifying communities based on the abundance of DNA copy numbers, and assuming a constant correlation factor between DNA content and biomass. Cho et al. ([2013]) determined DNA specific growth yields (DNA copy numbers/mg-N) but did not express results in terms of biomass. Ahn et al. ([2008]) used additional conversion factors to derive biomass growth yields (mg-COD biomass/mg-N) from growth yields expressed in terms of DNA. The prevalent use of DNA copy numbers (Cheng et al. [2011]; Chon et al. [2011]) or cell numbers (Coskuner et al. [2005]), rather than actual biomass, renders these results inconvenient for most engineering purposes. Error-prone conversion factors (e.g. gene copies/genome, genomes/cell, cells/g dry matter, DNA extraction efficiency) add to the problematic of this approach.

Several studies employed FISH to quantify cells of AOB and NOB, and to determine cell-specific ammonium and nitrite oxidation rates (Altmann et al. [2003]; Daims et al. [2001]; Gieseke et al. [2005]; Wagner et al. [1995]). In a direct comparison of FISH and qPCR for the quantification of cells in nitrifying biofilms Kindaichi et al. ([2006]) found that both methods yielded comparable results but qPCR was more favorable due to higher sensitivity and faster handling. Low sample concentrations (<10^5^ cells/mL), autofluorescence, non-specific binding and low signal intensity can become limiting factors for FISH analysis (Kindaichi et al. [2006]; Konuma et al. [2000]; Rittmann et al. [1999]; Zhang and Fang [2006]). The quantification of individual biomass concentrations by means of pyrosequencing remains challenging due to the semi-quantitative nature of the method (Amend et al. [2010]). Purely quantitative applications of pyrosequencing remain scarce as of yet. Lastly, biomass concentrations were estimated from observed substrate transformation rates, metabolite ratios and individual biomass growth yields (Jiang et al. [2011]; Lopez-Vazquez et al. [2007]; Rittmann et al. [1999]). These indirect methods are based on the measurement of commonly used analytical variables (e.g. substrate and product concentrations, lumped biomass concentration) without requiring molecular techniques (Lopez-Vazquez et al. [2007]). However, assumptions of reaction stoichiometry or maximum biomass specific conversion rates are inherent to these indirect methods and pose a major source of inaccuracy. In view of the previous, qPCR is regarded the most suited molecular method for the quantification of individual biomass concentrations in complex microbial ecosystems, and it stands out due to its high sensitivity (< 5 gene copies), high reproducibility (standard deviation < 2%) and high specificity (Kim et al. [2013]).

Here it is aimed to derive individual biomass concentrations, expressed in gram dry matter per liter, directly from the qPCR signal of a given sample. No such approach has been reported so far, despite a few key advantages: Firstly, the result can readily be used in mathematical models and engineering applications. Secondly, several limitations of existing methods can be avoided, including unnecessary assumptions or erroneous conversion factors.

## Material and methods

A defined coculture of *Desulfovibrio sp.* G11 and *Methanospirillum hungatei* JF1 was used to evaluate the applicability of a qPCR approach for the determination of specific biomass concentrations.

### Cultivation of microorganisms

Pure cultures of *Desulfovibrio sp.* strain G11 (DSM 7057) and *Methanospirillum hungatei* type strain JF1 (DSM 864) were obtained from the Laboratory of Microbiology, Wageningen University, The Netherlands, and cultivated in 2 L Schott bottles in the absence of oxygen and under sterile conditions. The basic medium was prepared according to Plugge ([2005]). Culture medium for *Desulfovibrio sp.* G11 contained 20 mM sodium lactate, as the sole carbon source, and 10 mM sodium sulphate as electron acceptor. Basic medium for *Methanospirillum hungatei* JF1 was supplemented with 2 mM sodium acetate and 4 mM cysteine hydrochloride. While *Desulfovibrio sp.* G11 was kept under 80%/20% N_2_/CO_2_ atmosphere, *Methanospirillum hungatei* JF1 was grown under 80%/20% H_2_/CO_2_. The headspace of the methanogenic culture was exchanged every other day. The pH was maintained between 7.0 and 7.2. All cultures were incubated at a temperature of 37 ºC and constantly shaken at 150 rpm.

### Centrifugation efficiency test

The centrifugation efficiency was tested for a biomass concentration of 21.0 mg/L (*Desulfovibrio sp.* G11) and 179.9 mg/L (*Methanospirillum hungatei* JF1) and four further two-fold dilutions, respectively. A three-step centrifugation procedure using a cell suspension volume of 2 mL (13000 rpm, 21,000 × g, 4ºC, Heraeus, Biofuge fresco) was used. The duration of the first step amounted to 5 min. The resulting supernatant was again centrifuged for 3 min. Supernatant of the second step was centrifuged for 10 min. Three pellets resulting from the previous centrifugation steps were combined for DNA extraction.

### DNA extraction

The UltraClean microbial DNA isolation kit (Mo Bio, Carlsbad, USA) was used for DNA extraction in triplicates. Instead of horizontal vortex mixing for 10 min, the Mini Bead Beater 16 (BioSpec Products, Bartlesville, USA) was used for 5 min. In order to improve DNA elution efficiency herring-sperm DNA (HS-DNA) was added prior to the bead-beating step.

### Quantitative PCR

Quantitative PCR was performed using an iQ5 system (Bio-Rad Laboratories B.V., Veenedaal, The Netherlands). The primer sets used for the amplification of the partial 16S rDNA sequences by qPCR are shown in Table 1. Both primer sets are highly specific to amplify only the DNA of the target microorganism in the coculture.

**Table 1:** Primer sets and sequences used for the amplification of the partial 16S rDNA sequences by qPCR

Primer name	Target	Primer sequence (5’-3’)	Reference
DSVsp G11 201f	*Desulfovibrio* sp. strain G11	GACCTCTGCTTGCATGTTACC	This study
DSVsp G11 471r		CTGATTAGCACAGTGCGGTTT	This study
Arch25f	Archaea	CTG GTT GAT CCT GCC AG	Mariakakis et al. ([2011])
MH236r	*Methanospirillum hungatei* JF1	CAG ACT CAT CCT GAA GCG AC	Worm et al. ([2011])

#### Primer efficiency

Specific primers for *Desulfovibrio* sp. G11 were designed using the ARB software program (Ludwig et al. [2004]). The optimum annealing temperature was determined using qPCR with a temperature gradient ranging from 50°C to 65°C. Primer efficiency was tested at the optimum annealing temperature using dilution series of the extracted genomic DNA in the range of 10^−1^ to 10^−6^. Each sample was analysed by qPCR in triplicate. From the exponential behaviour of the Ct value as a function of the DNA starting concentration, the primer efficiency was deduced. A combination of the specific primer MH236r and the general archaeal primer Arch25f was used as a primer set for *Methanospirillum hungatei* JF1. The optimum annealing temperature was determined using qPCR with a temperature gradient ranging from 52°C to 65°C. The qPCR amplification for *Desulfovibrio sp.* G11 proceeded according to the following scheme: 95°C (5 min), for the next 40 cycles 95°C (30 s), 62°C (40 s), 72°C (40 s), 80°C (25 s). The amplification for *Methanospirillum hungatei* JF1 was as follows: 95°C (5 min), for the next 40 cycles 95°C (30 s), 57°C (30 s), 72°C (15 s), 80°C (25 s). The bacterial reaction mixture consisted of 10 μl 2x iCycler mix (Bio-Rad, Hercules, USA), 0.1 μl DSVsp G11 201f primer (50 μM stock), 0.1 μl DSVsp G11 471r primer (50 μM stock), and 2.5 μl template DNA (5.0 ng/μl). In contrast, 0.2 μl Arch25f primer (50 μM stock) and 0.2 μl MH236r primer (50 μM stock) were used for the archaeal assay. Reaction mixtures were filled up to 20 μl with PCR-H_2_0.

#### Primer cross-sensitivity

To assess primer cross-sensitivity, qPCR was performed with the following primer/DNA mixtures: (1) DSVsp G11 201f and DSVsp G11 471r primer/HS-DNA and DNA of *Methanospirillum hungatei* JF1; (2) Arch25f and MH236r primer/HS-DNA and DNA of *Desulfovibrio sp.* G11.

### Determination of dry weight

Total suspended solids (TSS) of both pure cultures were determined according to standard filtration methods (Taras et al. [1971]). A Supor® 200 PES membrane filter with a pore size of 0.2 μm (PALL, Port Washington, USA) was used. The ash content was determined according to ESS Method 340.2 (WSLH [1993]). All measurements were conducted in triplicate. By subtracting the ash content from the TSS concentration, the concentration of volatile suspended solids (VSS) was obtained.

### Calibration curves

Pure cultures of *Desulfovibrio sp.* G11 and *Methanospirillum hungatei* JF1 were first diluted to a biomass concentration of 1.1 mg/L (*Desulfovibrio* sp. G11) and 9.0 mg/L (*Methanospirillum hungatei* JF1) and then combined to obtain co-cultures of known biomass ratios. The cell suspensions were taken from the late exponential/early stationary state. The optical density was measured at 660 nm (DR 2800, Hach-Lange, Tiel, The Netherlands) and was in the range of 0.150 and 0.250 for both cultures. Coculture samples (2 mL) of known biomass mixing ratios were centrifuged and the DNA was extracted according to the described procedures. At low DNA concentration of the sample, a volume of 5 μl of 10-fold diluted HS-DNA (10 μg/μl, Sigma) was added to improve the elution efficiency during DNA extraction. HS-DNA was added just before using the mini bead beater. To obtain a calibration curve relating the Ct value to the biomass concentration in the sample for each of the two species, qPCR was performed either with primers specific for *Desulfovibrio sp.* G11 or *Methanospirillum hungatei* JF1. All measurements were performed in triplicates. Instead of 2.5 μl of 5 ng/μl DNA template, 2.5 μl of 60-fold diluted DNA template was used for the qPCR.

## Results

**Figure 1:**
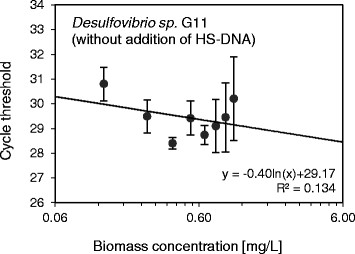
**qPCR calibration line of*Desulfovibrio sp.*** G11 obtained after DNA extraction in the absence of HS-DNA. The Ct values are plotted against the biomass concentration of *Desulfovibrio sp.* G11. The horizontal axis is displayed on a logarithmic scale. Vertical error bars were obtained from qPCR triplicates.

**Figure 2:**
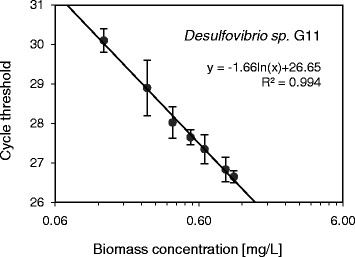
**qPCR calibration line of*Desulfovibrio sp.*** G11 in the presence of HS-DNA. The Ct values are plotted against the biomass concentration of *Desulfovibrio sp.* G11. The horizontal axis is displayed on a logarithmic scale. Vertical error bars were obtained from qPCR triplicates.

**Figure 3:**
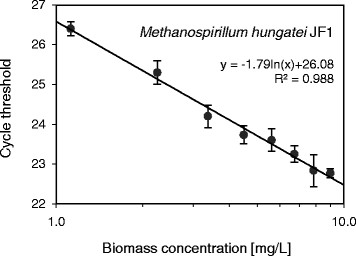
**qPCR calibration line of*Methanospirillum hungatei*JF1 in the presence of HS-DNA.** The Ct values are plotted against the biomass concentration of *Methanospirillum hungatei* JF1. The horizontal axis is displayed on a logarithmic scale. Vertical error bars were obtained from qPCR triplicates.

Initial attempts to establish calibration curves between the biomass concentration used and the resulting qPCR signal, using an unmodified standard procedure, yielded unsatisfactory results. Most notably fitted calibration curves of *Desulfovibrio sp.* G11 were of low quality (R^2^ = 0.134) and data generally suffered from high standard deviation (see Figure 1). Key steps of the calibration procedure were further investigated and optimized to obtain adequate calibration curves. The optimized qPCR calibration curves obtained for *Methanospirillum hungatei* JF1 and *Desulfovibrio sp.* G11 are presented in Figures 2 and 3, respectively. Results concerning the individual steps of the calibration procedure are shown below.

### Centrifugation efficiency

A low centrifugation efficiency leads to cell loss, thereby limiting the total amount of DNA available for qPCR, and fluctuations in centrifugation efficiency are reflected in the qPCR calibration curve. A three-step centrifugation procedure yielded best cell recovery with a centrifugation efficiency consistently above 97%. A standard error of 5% was derived from triplicate measurement.

### DNA extraction efficiency

HS-DNA was added during the DNA extraction step to enhance DNA elution from the filter membrane of the used DNA extraction kit. Leaving all other steps of the calibration procedure unchanged, the addition of HS-DNA during DNA extraction yielded significant improvements of the calibration curves. Largest improvements were obtained with *Desulfovibrio sp.* G11, as can be seen in Figure 1 (without HS-DNA) and Figure 2 (with HS-DNA). Calibration data obtained for *Desulfovibrio sp.* G11 in the presence of HS-DNA resulted in a more accurate regression line (R^2^ = 0.994) compared to the case without addition of HS-DNA (R^2^ = 0.134).

To confirm a constant DNA extraction efficiency, DNA was extracted from biomass samples of different concentrations and subjected to qPCR. Taking primer efficiency and the dilution factor between samples into account the expected theoretical difference in Ct values with regard to the undiluted sample can be calculated. It was found that experimental and theoretical Ct values differed on average by less than 0.9% for *Methanospirillum hungatei* JF1, and less than 1.2% for *Desulfovibrio sp.* G11 in the presence of HS-DNA. In contrast, the same difference amounted to 7.5% for *Desulfovibrio sp.* G11 in the absence of HS-DNA. These results confirm that a constant DNA extraction efficiency was achieved for both species irrespective of the biomass starting concentration due to the addition of HS-DNA.

### Quantitative PCR

#### Primer efficiency

A primer efficiency of 93.4% was obtained at an optimum annealing temperature of 57°C for *Methanospirillum hungatei* JF1. The primer efficiency for *Desulfovibrio sp.* G11 was 75.9% at 62°C. Primer efficiency was constant and remained unaffected by the presence of HS-DNA.

#### Primer cross-sensitivity

No significant primer cross-sensitivities were observed in the calibrated biomass concentration range. Cross-sensitivities occurred at biomass concentrations 100-fold lower than the minimum biomass concentrations used for the preparation of the calibration lines.

#### qPCR accuracy

The Ct values obtained from qPCR triplicates of *Methanospirillum hungatei* JF1 showed a standard deviation of 1.6% and 2.7% for *Desulfovibrio sp.* G11.

### Determination of dry weight

The measurement error of the VSS determination is 1.8% (*Methanospirillum hungatei* JF1) and 0.6% (*Desulfovibrio* sp. G11).

## Discussion

Seitz et al. ([1990]) made the first attempt to determine individual biomass concentrations in methanogenic microbial cocultures by manual cell counting. This approach is not only tedious but is also affected by varying cell morphologies and the occurrence of cell aggregates which renders cell counting less accurate than DNA-based techniques. In this study qPCR was investigated for the determination of individual biomass concentrations because of its reported higher accuracy, sensitivity and reproducibility compared to other quantification methods such as quantitative FISH or pyrosequencing (Amend et al. [2010]; Kim et al. [2013]).

The present study successfully demonstrated the suitability of a newly developed qPCR approach for the quantification of individual biomass concentrations in a defined methanogenic coculture of *Desulfovibrio sp.* G11 and *Methanospirillum hungatei* JF1. Calibration curves correlating the native qPCR signal (Ct value) directly with absolute biomass concentrations (mg dry matter/L) were obtained with high accuracy. A biomass calibration curve was established in a concentration range of 1.1 – 9.0 mg/L for *Methanospirillum hungatei* JF1 and 0.1 – 1.1 mg/L for *Desulfovibrio sp.* G11. Dilution or pre-concentration of samples can be used to ensure that biomass concentrations are within the calibrated regime. The calibrated biomass concentration range can possibly be extended much further into both directions given that qPCR has a reported dynamic range of more than 6 orders of magnitude (Kim et al. [2013]). However, in practice, saturation of the DNA extraction kit (at about 10^9^ cells/mL) may pose an upper detection limit for the presented approach and sample dilution will be required (data not shown). For this reason it is advisable to use 10 mg/L (*Desulfovibrio* sp. G11) and 100 mg/L (for *Methanospirillum hungatei* JF1) as the respective upper limits (data not shown) which roughly equals 10^8^ cells/mL. The lower detection limit is most likely determined by DNA elution efficiency and primer cross-sensitivity. Addition of HS-DNA during the DNA extraction step increased the DNA elution efficiency significantly, most notably for *Desulfovibrio* sp. G11, while showing no noticeable cross-sensitivities with the employed primer pairs.

Many qPCR approaches for the quantification of microbial communities rely on the knowledge of the DNA extraction efficiency, the number of gene copies per genome and the number of genome copies per organism. However, such data is not always available in genomic databases and may show broad variation (Kim et al. [2013]; Malandrin et al. [1999]; Mileyko et al. [2008]). Another factor of uncertainty is a varying DNA extraction efficiency. As a result, the conversion of DNA copy numbers to actual biomass concentrations is often a vague procedure. The here presented quantification method overcomes these limitations because the native qPCR signal is directly linked to the biomass concentration of the species of interest. The aforementioned conversion factors are not required and the DNA extraction efficiency needs not to be known explicitly. Nevertheless, it must be ensured that DNA extraction efficiency is constant between replicates. A constant extraction efficiency was confirmed for the target organisms in this study.

Suitability of the presented qPCR approach for the determination of individual biomass concentrations in a defined coculture of *Desulfovibrio sp.* G11 and *Methanospirillum hungatei* JF1 was successfully demonstrated in this study. Feasibility of the proposed method in non-defined environmental samples was not yet examined. High primer specificity is required in non-defined samples. The newly designed primer set for *Desulfovibrio sp.* G11 certainly meets that criterion. In contrast, the primer set used for DNA amplification of *Methanospirillum hungatei* JF1 consists of an archaea-specific forward primer and a reverse primer highly specific for *Methanospirillum hungatei* JF1. The analysis of non-defined environmental samples may require a species specific reverse primer to ensure only *Methanospirillum hungatei* JF1 is detected.

Group-specific determination of biomass concentrations remains challenging with this method. In principle all species belonging to the microbial group in question have to be grown in pure culture for the preparation of single-species calibration curves. Summation of species-specific biomass concentrations of an environmental sample, derived from the respective calibration curves, yields the biomass concentration of a desired group consisting of the different species previously assigned to it. This procedure is very time-consuming but it remains a one-time activity. Apart from that, in non-defined microbial communities target organisms need to be identified first (e.g. by denaturing gradient gel electrophoresis) to assign them to the microbial group of interest.

Future research should aim for a comparison of the presented qPCR approach with other existing methods such as qFISH or pyrosequencing, especially with regard to environmental samples. While the authors believe that the presented qPCR approach shows its specific qualities in the accurate quantification of individual biomass concentrations of single species, it is recognized that FISH and pyrosequencing can serve quite complementary purposes: Solely FISH is capable to identify cell distribution and cell interaction in-situ (Kindaichi et al. [2007]; Okabe et al. [1999]), while pyrosequencing is regarded most suited for the high-throughput analysis of complex non-defined microbial communities (Amend et al. [2010]; Ronaghi and Elahi [2002]).

The proposed quantification method poses a major improvement over prevailing approaches because no error-prone conversion factors and assumptions are needed to obtain absolute biomass concentrations. The method developed is therefore ideally suited for engineering applications and for providing model input. The gained knowledge on individual biomass concentrations in defined or non-defined microbial communities opens up the opportunity to calculate biomass specific conversion rates which enables the validation of models for anaerobic digestion processes and other mixed microbial conversions.

## Competing interests

The authors declare that they have no competing interests.
